# Can functional magnetic resonance imaging provide new mechanistic and prognostic insight following neonatal hypoxic‐ischaemic encephalopathy?

**DOI:** 10.1111/dmcn.16392

**Published:** 2025-06-27

**Authors:** Emil Galanides, Jonathan O'Muircheartaigh, Tomoki Arichi

**Affiliations:** ^1^ MRC Centre for Neurodevelopmental Disorders King's College London London UK

## Abstract

**This commentary is on the original article by Håkansson et al. on pages** 99–109 **of this issue.**

Hypoxic‐ischaemic encephalopathy (HIE) has long been a major global cause of neonatal death and childhood disability. To date, the only available treatment for moderate to severe HIE is therapeutic hypothermia within the first few hours after birth, which has been proven in clinical trials to reduce the risk of major disability and death.^1^ Despite this, a considerable number of infants will still go on to develop neurodevelopmental difficulties including cognitive impairment. The broad range of potential outcomes from HIE presents paediatricians with a complex challenge when counselling parents and planning provision of subsequent support through childhood. Part of this challenge includes our lack of mechanistic understanding about the pathophysiology underlying long‐term neurological impairment following neonatal injury. Therefore, there is a great need for predictive biomarkers of motor and cognitive ability.

Advances in non‐invasive magnetic resonance imaging (MRI) techniques applied to paediatric care have provided marked new mechanistic insights into typical and atypical functional development following brain injury. By measuring temporal changes in regional brain blood oxygen delivery, functional MRI (fMRI) provides an indirect approximation of neuronal activity during a task or at rest. In adults at rest, fMRI reveals a consistent set of functionally connected networks that reflect both somatomotor processing and higher order associative networks linked to cognition. Over functional brain development, these networks have been shown to refine and strengthen their connections, with disruption to this process associated with atypical development.^2^

Håkansson et al. leverage resting state fMRI and motor performance tests to investigate the impact of HIE in later childhood.^3^ They identify deficits in multiple motor domains that were associated with correlation strengths between somatomotor and some association networks. This is of particular interest, as the changes in functional connectivity likely reflect compensatory or pathological functional reorganization resulting from hypoxic injury. Their study adds valuable insight into the long‐term functional outcomes of HIE, a key concern for parents and caregivers, by supporting the evidence of late childhood motor deficits even without a diagnosis of cerebral palsy.^4^ More broadly, as all children in the study received therapeutic hypothermia, it could also support the need for adjunctive or synergistic therapies to improve outcomes.

This study highlights a number of issues which make generalization of study findings in this field challenging. First, due to the relative infrequency of the condition in high‐income countries; second, studying subsets of, for example, severity, treatment, and outcome in an intrinsically heterogeneous clinical population; and third, loss or reduced quality of MRI due to excessive motion, especially when scanning children.

Therefore, it is important to consider accessibility and standardization in the research of HIE therapies and biomarkers, to ensure they are both available and practical for routine use in low‐ and middle‐income countries where HIE has the greatest impact, yet specialist healthcare – including advanced MRI facilities and trained staff – may not be widely available. Feasibility assessments and validation of research findings in these populations are key to addressing this.

Furthermore, the majority of HIE studies focus on moderate to severe cases, despite mild cases – which are not currently indicated for therapeutic hypothermia – also being likely associated with an increased risk of later developmental impairment. It would be interesting to investigate whether the same functional connectivity observed by Håkansson et al. is evident in such cases – and crucially, how it changes in trials of novel neuroprotective treatments, where these long‐term outcome measures are urgently needed.^5^ Together, an understanding of the entire spectrum of HIE outcomes with affordable prognostic methods and treatments would address this major global health issue.

## Data Availability

Not required.
